# 132. “Diagnostic and Antibiotic Stewardship based on Urine Culture”

**DOI:** 10.1093/ofid/ofad500.205

**Published:** 2023-11-27

**Authors:** Nitheeshwar C, Mithra Priya Tharsini, Suresh Kumar Dorairajan

**Affiliations:** Jaya college of paramedical science, chennai, Tamil Nadu, India; Jaya college of paramedical science and, Chennai, Tamil Nadu, India; Apollo hospital, Chennai, Tamil Nadu, India

## Abstract

**Background:**

Asymptomatic bacteriuria is one of the most common causes of antibiotic use in hospitalised patients. Antibiotic stewardship targeting asymptomatic bacteriuria ( ASB) should focus on diagnostic urine cultures, restricting unnecessary urine cultures, and preventing inappropriate antibiotic use. However, there is limited evidence from developing countries on the impact of the above interventions. This study aimed to assess the appropriateness of urine cultures ordered and also study the antibiotic prescribing pattern for the patient who underwent urine cultures.

**Methods:**

A prospective, observational study was conducted in a tertiary care hospital in south India between February 2023 and April 2023. The medical records of the hospitalised patients who underwent urine culture ( except urological and pregnant women) were reviewed by using the validated audit tool, which contained details of demographic data, indications, results of urine culture, and antibiotics prescribed to the patients. The data was audited by the trained Pharm.D. students to establish the appropriateness of the urine cultures ordered and the antibiotic prescribing pattern for the patients who started antibiotics.

**Results:**

During the study period 329 case records were audited. And the data is given in the table 1.

**Table 1:**
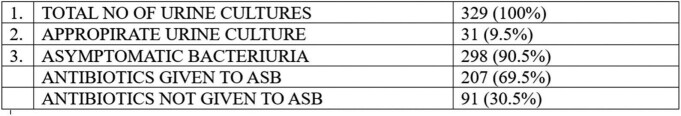
Diagnostic stewardship and antibiotic prescribing practices

**Conclusion:**

Most of the urine cultures ( 90.5%) sent are without any urinary symptoms, and 69.5% of patients with asymptomatic bacteriuria receive antibiotics, which contribute to antibiotic resistance. It is necessary to put effective techniques into practice to increase the appropriateness of ordering urine cultures and the use of antibiotics in hospitals.

**Disclosures:**

**All Authors**: No reported disclosures

